# ADVANCING AGE-FRIENDLY CARE IN THE VA: MIXED-METHODS IMPLEMENTATION RESULTS OF 39 IMPLEMENTATION INSTANCES

**DOI:** 10.1093/geroni/igae098.4362

**Published:** 2024-12-31

**Authors:** Amanda Peeples, LauraEllen Ashcraft, Octavia Goodman, Phoebe Whiteside, Rebecca Brown, Robert Burke, Connor Warren, Lindsay Pelcher

**Affiliations:** Corporal Michael J. Crescenz VA Medical Center, Philadelphia, Pennsylvania, United States; Corporal Michael J. Crescenz VA Medical Center, Philadelphia, Pennsylvania, United States; Corporal Michael J. Crescenz VA Medical Center, Philadelphia, Pennsylvania, United States; Corporal Michael J. Crescenz VA Medical Center, Philadelphia, Pennsylvania, United States; University of Pennsylvania, Philadelphia, Pennsylvania, United States; XXX; Corporal Michael J. Crescenz VA Medical Center, Philadelphia, Pennsylvania, United States; Veterans Health Administration, Pittsburgh, Pennsylvania, United States

## Abstract

Age-friendly care integrates the 4Ms (what Matters, Medication, Mentation, Mobility) to promote evidence-based care for older adults. The Safer Aging through Geriatrics-Informed Evidence-Based Practices Quality Enhancement Research Initiative (SAGE QUERI) is a hybrid type-III implementation-effectiveness trial comparing an implementation strategy bundle to dissemination as usual. We implemented four evidence-based practices (EBPs) aligned with the 4Ms across 9 VA Medical Centers. We used descriptive statistics and qualitative interviews with 30 implementing clinicians to report mixed-methods outcomes for reach, adoption, implementation, and maintenance across 39 implementation instances. Reach to Veterans varied widely across EBPs: Mobility n=30; Mentation n=37; Medication n=184; what Matters n=8000+. Eligibility criteria impacted reach; clinicians reported the ease of enrolling Veterans in an EBP with wide eligibility (e.g., every Veteran who is a candidate for surgery) versus an EBP with strict eligibility (e.g., “all the moons have to be aligned” to identify/enroll Veterans). Across 39 implementation instances, 77% (n=31) initially adopted the EBP, with a total of 169 clinicians trained (what Matters n=21; Mobility n=29; Medication n=30; Mentation n=89). Clinicians described training requirements, alignment of EBPs with current practices, and organizational support as key factors influencing adoption. Implementation fidelity varied by EBP: 34% what Matters; 80% Mobility; 100% Mentation and Medication. Clinicians noted the wider impact of EBP-related practice changes, including using new skills with Veterans ineligible for the full EBPs. Across 39 implementation instances, 51% (n=20) reached full maintenance; an additional 23% (n=9) achieved implementation. Sites that had successfully integrated EBPs into practice expressed intentions to sustain EBPs.

